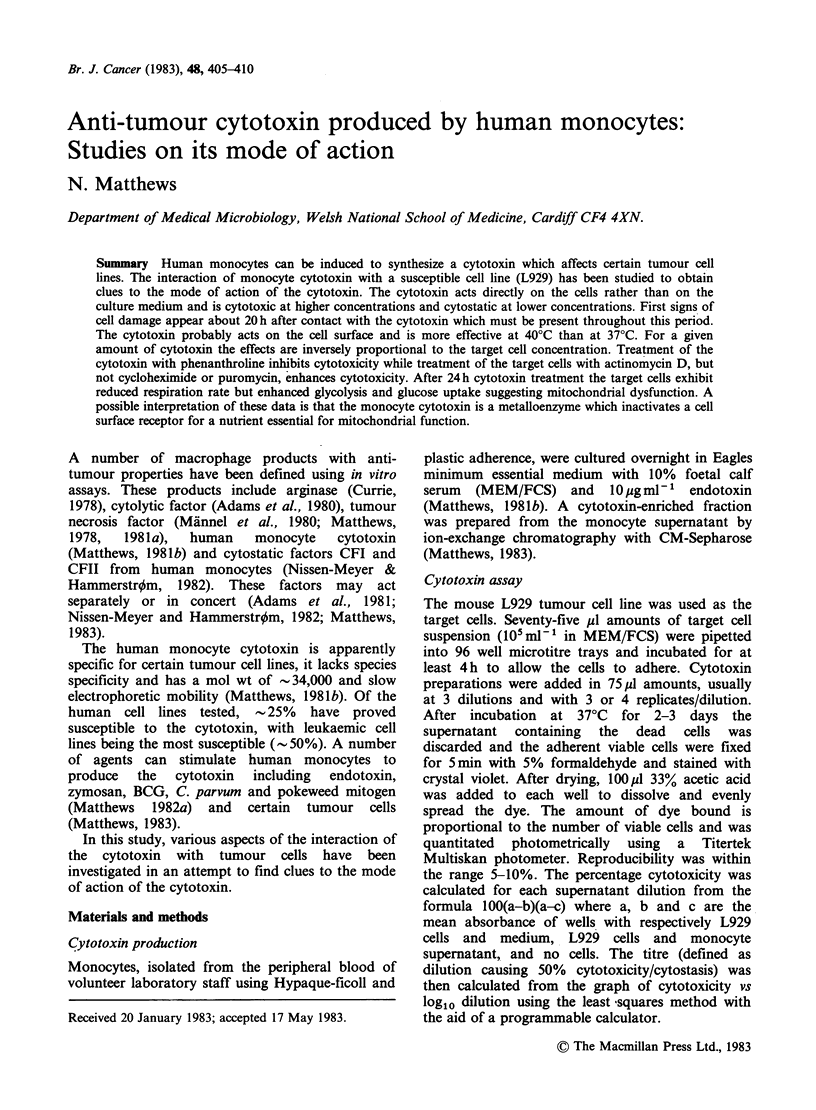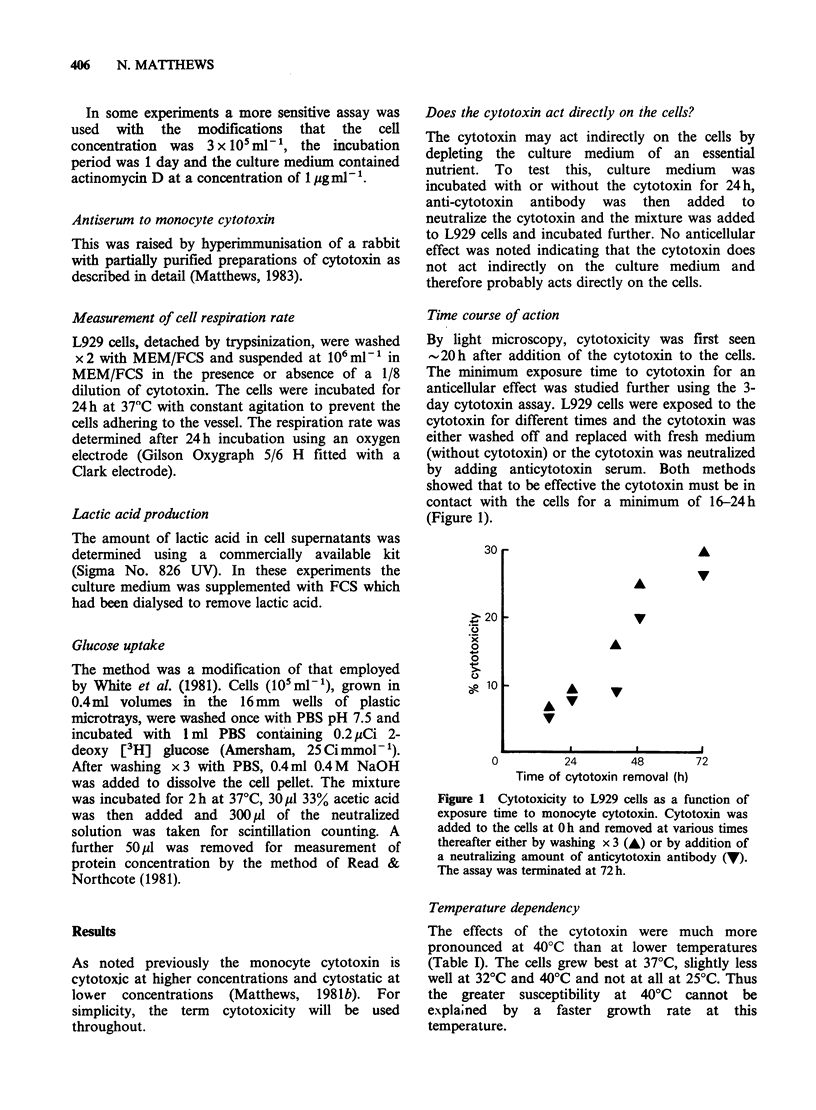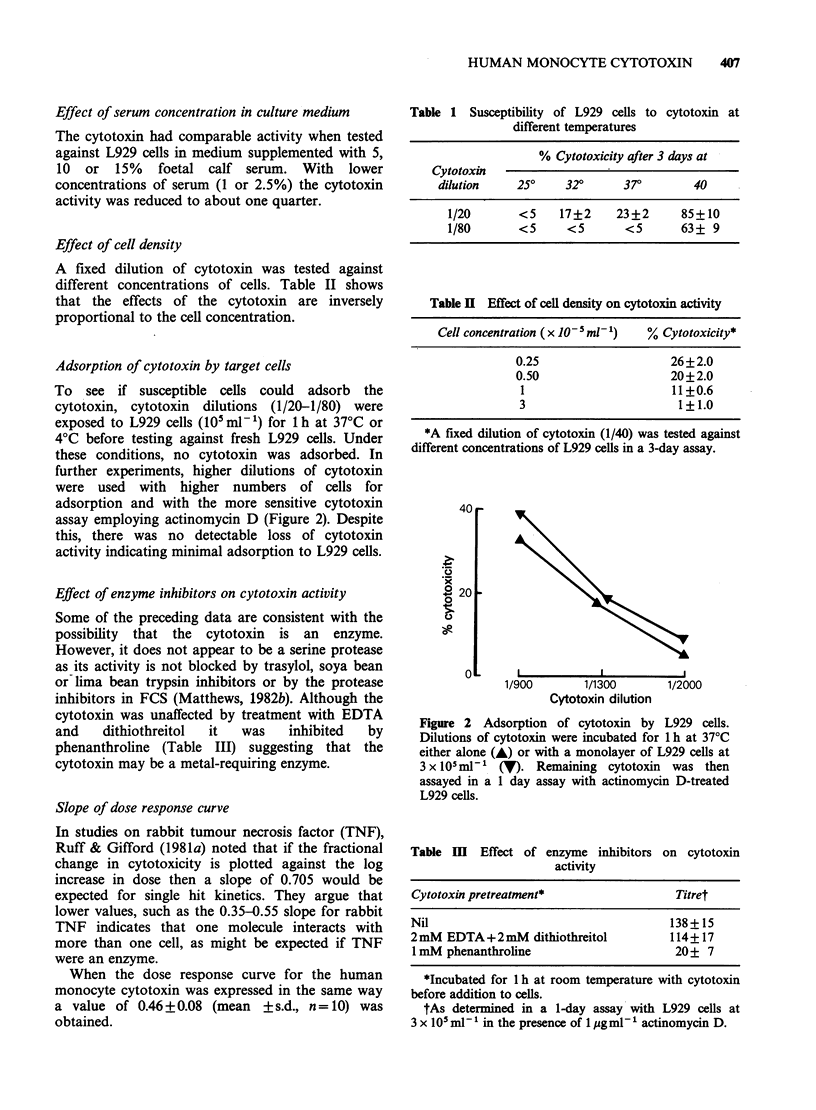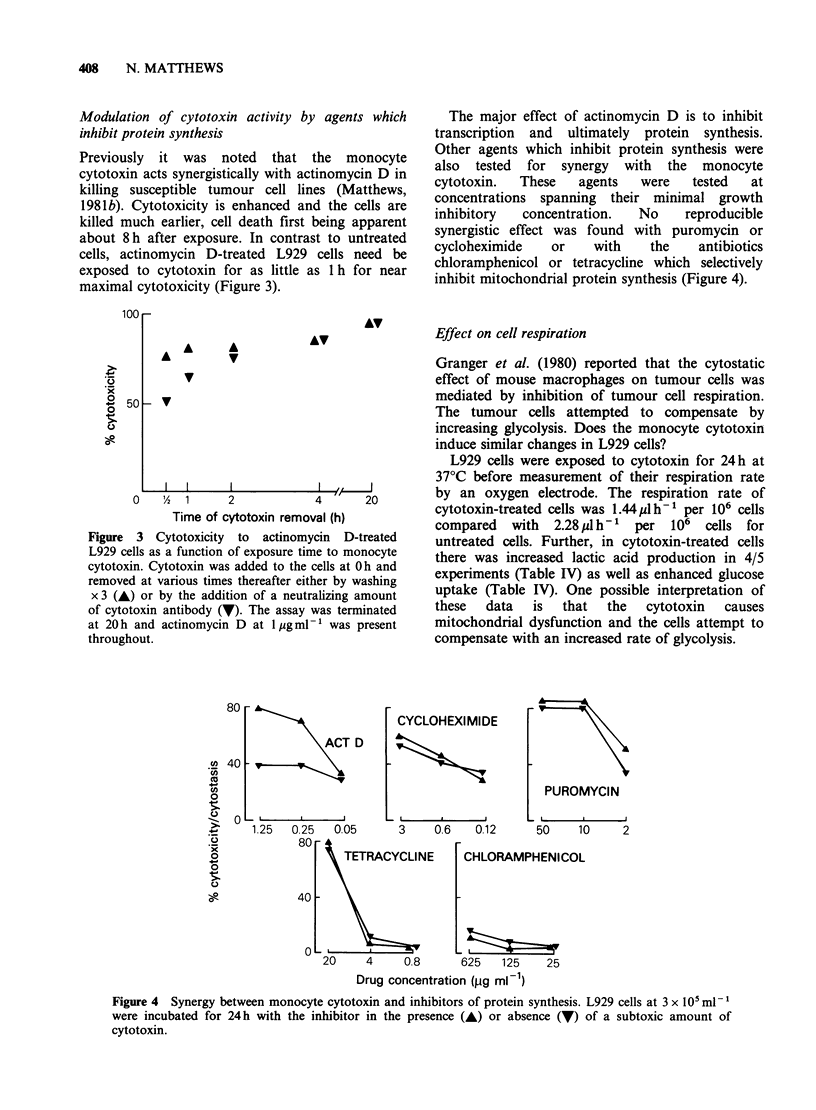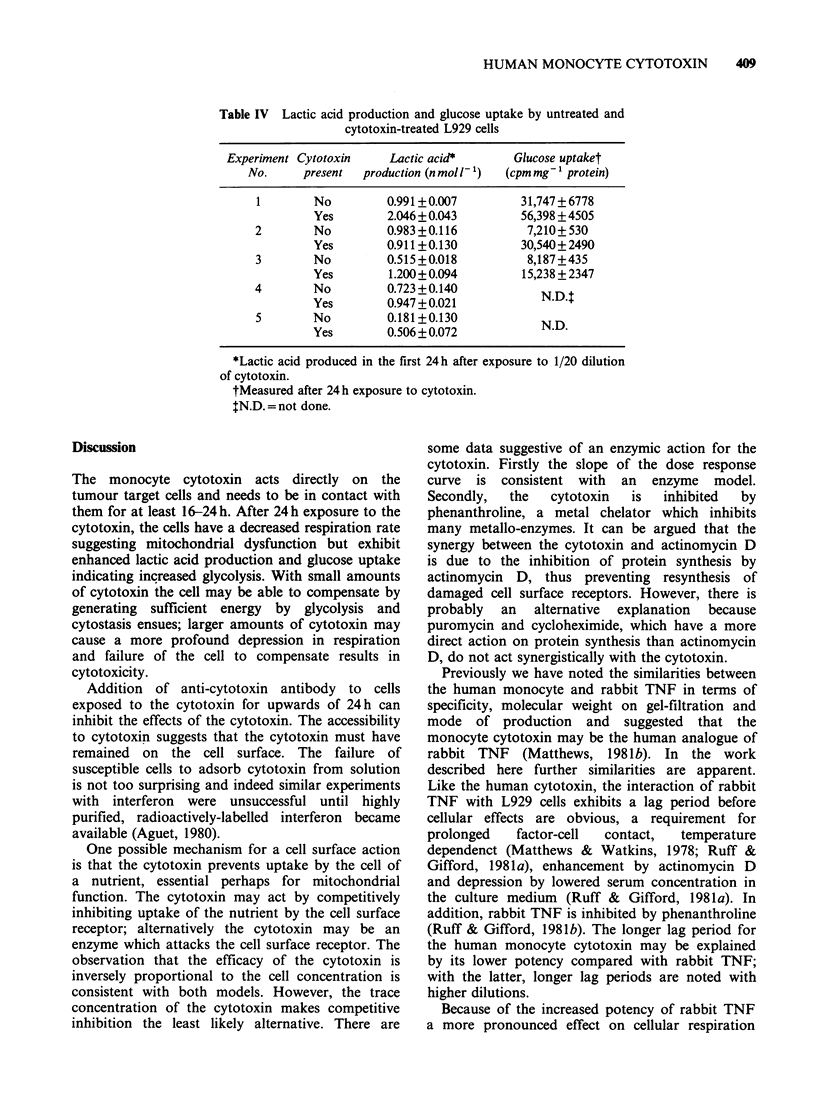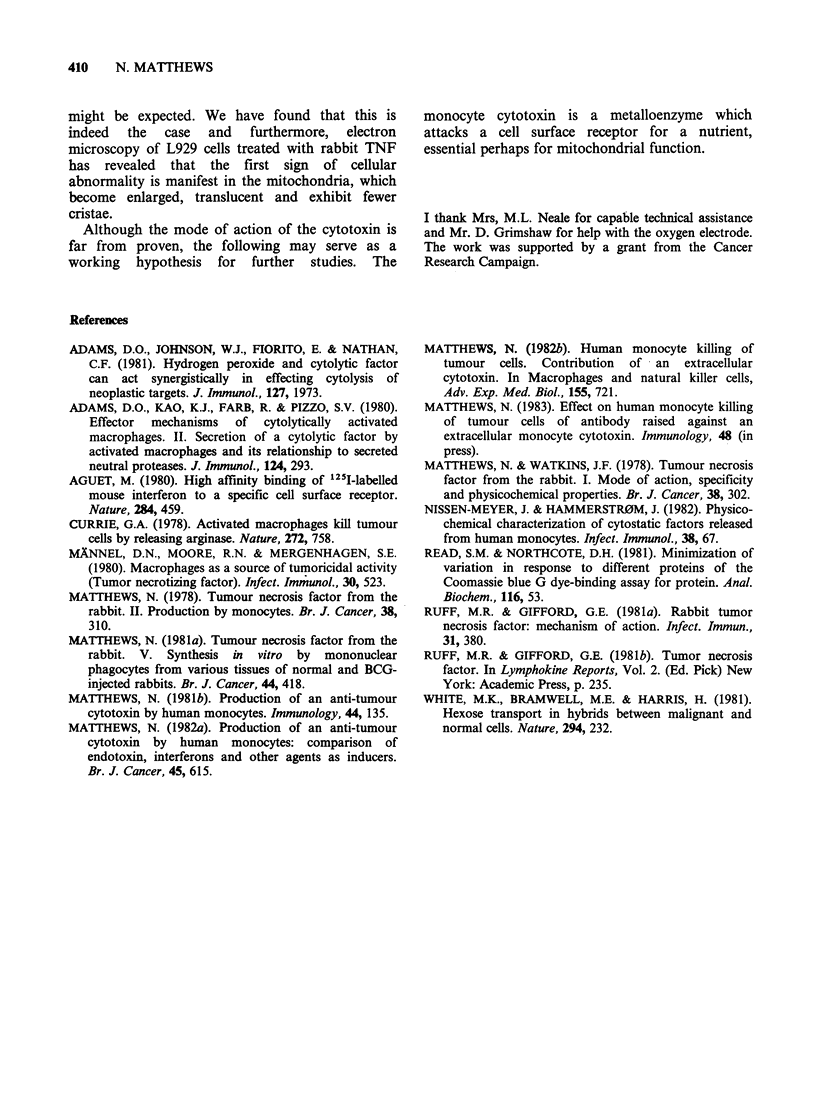# Anti-tumour cytotoxin produced by human monocytes: studies on its mode of action.

**DOI:** 10.1038/bjc.1983.205

**Published:** 1983-09

**Authors:** N. Matthews

## Abstract

Human monocytes can be induced to synthesize a cytotoxin which affects certain tumour cell lines. The interaction of monocyte cytotoxin with a susceptible cell line (L929) has been studied to obtain clues to the mode of action of the cytotoxin. The cytotoxin acts directly on the cells rather than on the culture medium and is cytotoxic at higher concentrations and cytostatic at lower concentrations. First signs of cell damage appear about 20 h after contact with the cytotoxin which must be present throughout this period. The cytotoxin probably acts on the cell surface and is more effective at 40 degrees C than at 37 degrees C. For a given amount of cytotoxin the effects are inversely proportional to the target cell concentration. Treatment of the cytotoxin with phenanthroline inhibits cytotoxicity while treatment of the target cells with actinomycin D, but not cycloheximide or puromycin, enhances cytotoxicity. After 24 h cytotoxin treatment the target cells exhibit reduced respiration rate but enhanced glycolysis and glucose uptake suggesting mitochondrial dysfunction. A possible interpretation of these data is that the monocyte cytotoxin is a metalloenzyme which inactivates a cell surface receptor for a nutrient essential for mitochondrial function.


*Br. J. Cancer* (1983), **48**, 405–410

### Anti-tumour cytotoxin produced by human monocytes: Studies on its mode of action

N. Matthews

*Department of Medical Microbiology, Welsh National School of Medicine, Cardiff CF4 4XN.*

**Summary** Human monocytes can be induced to synthesize a cytotoxin which affects certain tumour cell lines. The interaction of monocyte cytotoxin with a susceptible cell line (L929) has been studied to obtain clues to the mode of action of the cytotoxin. The cytotoxin acts directly on the cells rather than on the culture medium and is cytotoxic at higher concentrations and cytostatic at lower concentrations. First signs of cell damage appear about 20 h after contact with the cytotoxin which must be present throughout this period. The cytotoxin probably acts on the cell surface and is more effective at 40°C than at 37°C. For a given amount of cytotoxin the effects are inversely proportional to the target cell concentration. Treatment of the cytotoxin with phenanthroline inhibits cytotoxicity while treatment of the target cells with actinomycin D, but not cycloheximide or puromycin, enhances cytotoxicity. After 24 h cytotoxin treatment the target cells exhibit reduced respiration rate but enhanced glycolysis and glucose uptake suggesting mitochondrial dysfunction. A possible interpretation of these data is that the monocyte cytotoxin is a metalloenzyme which inactivates a cell surface receptor for a nutrient essential for mitochondrial function.

A number of macrophage products with anti-tumour properties have been defined using *in vitro* assays. These products include arginase (Currie, 1978), cytolytic factor (Adams *et al.*, 1980), tumour necrosis factor (Männel *et al.*, 1980; Matthews, 1978, 1981*a*), human monocyte cytotoxin (Matthews, 1981*b*) and cytostatic factors CFI and CFII from human monocytes (Nissen-Meyer & Hammerstrøm, 1982). These factors may act separately or in concert (Adams *et al.*, 1981; Nissen-Meyer and Hammerstrøm, 1982; Matthews, 1983).

The human monocyte cytotoxin is apparently specific for certain tumour cell lines, it lacks species specificity and has a mol wt of ~34,000 and slow electrophoretic mobility (Matthews, 1981*b*). Of the human cell lines tested, ~25% have proved susceptible to the cytotoxin, with leukaemic cell lines being the most susceptible (~50%). A number of agents can stimulate human monocytes to produce the cytotoxin including endotoxin, zymosan, BCG, *C. parvum* and pokeweed mitogen (Matthews 1982*a*) and certain tumour cells (Matthews, 1983).

In this study, various aspects of the interaction of the cytotoxin with tumour cells have been investigated in an attempt to find clues to the mode of action of the cytotoxin.

### Materials and methods

### *Cytotoxin production*

Monocytes, isolated from the peripheral blood of volunteer laboratory staff using Hypaque-ficoll and plastic adherence, were cultured overnight in Eagles minimum essential medium with 10% foetal calf serum (MEM/FCS) and 10 $\mu g\, ml^{-1}$ endotoxin (Matthews, 1981*b*). A cytotoxin-enriched fraction was prepared from the monocyte supernatant by ion-exchange chromatography with CM-Sepharose (Matthews, 1983).

### *Cytotoxin assay*

The mouse L929 tumour cell line was used as the target cells. Seventy-five $\mu l$ amounts of target cell suspension ($10^5\, ml^{-1}$ in MEM/FCS) were pipetted into 96 well microtitre trays and incubated for at least 4 h to allow the cells to adhere. Cytotoxin preparations were added in 75 $\mu l$ amounts, usually at 3 dilutions and with 3 or 4 replicates/dilution. After incubation at 37°C for 2–3 days the supernatant containing the dead cells was discarded and the adherent viable cells were fixed for 5 min with 5% formaldehyde and stained with crystal violet. After drying, 100 $\mu l$ 33% acetic acid was added to each well to dissolve and evenly spread the dye. The amount of dye bound is proportional to the number of viable cells and was quantitated photometrically using a Titertek Multiskan photometer. Reproducibility was within the range 5–10%. The percentage cytotoxicity was calculated for each supernatant dilution from the formula 100(a–b)(a–c) where a, b and c are the mean absorbance of wells with respectively L929 cells and medium, L929 cells and monocyte supernatant, and no cells. The titre (defined as dilution causing 50% cytotoxicity/cytostasis) was then calculated from the graph of cytotoxicity *vs* $\log_{10}$ dilution using the least squares method with the aid of a programmable calculator.

Received 20 January 1983; accepted 17 May 1983.

© The Macmillan Press Ltd., 1983

In some experiments a more sensitive assay was used with the modifications that the cell concentration was $3 \times 10^5\,ml^{-1}$, the incubation period was 1 day and the culture medium contained actinomycin D at a concentration of $1\,\mu g\,ml^{-1}$.

*Antiserum to monocyte cytotoxin*

This was raised by hyperimmunisation of a rabbit with partially purified preparations of cytotoxin as described in detail (Matthews, 1983).

*Measurement of cell respiration rate*

L929 cells, detached by trypsinization, were washed $\times 2$ with MEM/FCS and suspended at $10^6\,ml^{-1}$ in MEM/FCS in the presence or absence of a 1/8 dilution of cytotoxin. The cells were incubated for 24 h at 37°C with constant agitation to prevent the cells adhering to the vessel. The respiration rate was determined after 24 h incubation using an oxygen electrode (Gilson Oxygraph 5/6 H fitted with a Clark electrode).

*Lactic acid production*

The amount of lactic acid in cell supernatants was determined using a commercially available kit (Sigma No. 826 UV). In these experiments the culture medium was supplemented with FCS which had been dialysed to remove lactic acid.

*Glucose uptake*

The method was a modification of that employed by White *et al.* (1981). Cells ($10^5\,ml^{-1}$), grown in 0.4 ml volumes in the 16 mm wells of plastic microtrays, were washed once with PBS pH 7.5 and incubated with 1 ml PBS containing $0.2\,\mu Ci$ 2-deoxy [$^3H$] glucose (Amersham, $25\,Ci\,mmol^{-1}$). After washing $\times 3$ with PBS, 0.4 ml 0.4 M NaOH was added to dissolve the cell pellet. The mixture was incubated for 2 h at 37°C, $30\,\mu l$ 33% acetic acid was then added and $300\,\mu l$ of the neutralized solution was taken for scintillation counting. A further $50\,\mu l$ was removed for measurement of protein concentration by the method of Read & Northcote (1981).

### Results

As noted previously the monocyte cytotoxin is cytotoxic at higher concentrations and cytostatic at lower concentrations (Matthews, 1981*b*). For simplicity, the term cytotoxicity will be used throughout.

*Does the cytotoxin act directly on the cells?*

The cytotoxin may act indirectly on the cells by depleting the culture medium of an essential nutrient. To test this, culture medium was incubated with or without the cytotoxin for 24 h, anti-cytotoxin antibody was then added to neutralize the cytotoxin and the mixture was added to L929 cells and incubated further. No anticellular effect was noted indicating that the cytotoxin does not act indirectly on the culture medium and therefore probably acts directly on the cells.

*Time course of action*

By light microscopy, cytotoxicity was first seen ~20 h after addition of the cytotoxin to the cells. The minimum exposure time to cytotoxin for an anticellular effect was studied further using the 3-day cytotoxin assay. L929 cells were exposed to the cytotoxin for different times and the cytotoxin was either washed off and replaced with fresh medium (without cytotoxin) or the cytotoxin was neutralized by adding anticytotoxin serum. Both methods showed that to be effective the cytotoxin must be in contact with the cells for a minimum of 16–24 h (Figure 1).

**Figure 1** Cytotoxicity to L929 cells as a function of exposure time to monocyte cytotoxin. Cytotoxin was added to the cells at 0 h and removed at various times thereafter either by washing × 3 (▲) or by addition of a neutralizing amount of anticytotoxin antibody (▼). The assay was terminated at 72 h.

*Temperature dependency*

The effects of the cytotoxin were much more pronounced at 40°C than at lower temperatures (Table I). The cells grew best at 37°C, slightly less well at 32°C and 40°C and not at all at 25°C. Thus the greater susceptibility at 40°C cannot be explained by a faster growth rate at this temperature.

*Effect of serum concentration in culture medium*

The cytotoxin had comparable activity when tested against L929 cells in medium supplemented with 5, 10 or 15% foetal calf serum. With lower concentrations of serum (1 or 2.5%) the cytotoxin activity was reduced to about one quarter.

*Effect of cell density*

A fixed dilution of cytotoxin was tested against different concentrations of cells. Table II shows that the effects of the cytotoxin are inversely proportional to the cell concentration.

*Adsorption of cytotoxin by target cells*

To see if susceptible cells could adsorb the cytotoxin, cytotoxin dilutions (1/20–1/80) were exposed to L929 cells ($10^5\,ml^{-1}$) for 1 h at 37°C or 4°C before testing against fresh L929 cells. Under these conditions, no cytotoxin was adsorbed. In further experiments, higher dilutions of cytotoxin were used with higher numbers of cells for adsorption and with the more sensitive cytotoxin assay employing actinomycin D (Figure 2). Despite this, there was no detectable loss of cytotoxin activity indicating minimal adsorption to L929 cells.

*Effect of enzyme inhibitors on cytotoxin activity*

Some of the preceding data are consistent with the possibility that the cytotoxin is an enzyme. However, it does not appear to be a serine protease as its activity is not blocked by trasylol, soya bean or lima bean trypsin inhibitors or by the protease inhibitors in FCS (Matthews, 1982*b*). Although the cytotoxin was unaffected by treatment with EDTA and dithiothreitol it was inhibited by phenanthroline (Table III) suggesting that the cytotoxin may be a metal-requiring enzyme.

*Slope of dose response curve*

In studies on rabbit tumour necrosis factor (TNF), Ruff & Gifford (1981*a*) noted that if the fractional change in cytotoxicity is plotted against the log increase in dose then a slope of 0.705 would be expected for single hit kinetics. They argue that lower values, such as the 0.35–0.55 slope for rabbit TNF indicates that one molecule interacts with more than one cell, as might be expected if TNF were an enzyme.

When the dose response curve for the human monocyte cytotoxin was expressed in the same way a value of $0.46 \pm 0.08$ (mean $\pm$s.d., $n=10$) was obtained.

**Table I** Susceptibility of L929 cells to cytotoxin at different temperatures

| *Cytotoxin dilution* | *% Cytotoxicity after 3 days at* 25° | 32° | 37° | 40 |
|---|---|---|---|---|
| 1/20 | <5 | 17±2 | 23±2 | 85±10 |
| 1/80 | <5 | <5 | <5 | 63± 9 |

**Table II** Effect of cell density on cytotoxin activity

| *Cell concentration* ($\times 10^{-5}\,ml^{-1}$) | *% Cytotoxicity** |
|---|---|
| 0.25 | 26±2.0 |
| 0.50 | 20±2.0 |
| 1 | 11±0.6 |
| 3 | 1±1.0 |

*A fixed dilution of cytotoxin (1/40) was tested against different concentrations of L929 cells in a 3-day assay.

**Figure 2** Adsorption of cytotoxin by L929 cells. Dilutions of cytotoxin were incubated for 1 h at 37°C either alone (▲) or with a monolayer of L929 cells at $3 \times 10^5\,ml^{-1}$ (▼). Remaining cytotoxin was then assayed in a 1 day assay with actinomycin D-treated L929 cells.

**Table III** Effect of enzyme inhibitors on cytotoxin activity

| *Cytotoxin pretreatment** | *Titre†* |
|---|---|
| Nil | 138±15 |
| 2 mM EDTA + 2 mM dithiothreitol | 114±17 |
| 1 mM phenanthroline | 20± 7 |

*Incubated for 1 h at room temperature with cytotoxin before addition to cells.

†As determined in a 1-day assay with L929 cells at $3 \times 10^5\,ml^{-1}$ in the presence of $1\,\mu g\,ml^{-1}$ actinomycin D.

*Modulation of cytotoxin activity by agents which inhibit protein synthesis*

Previously it was noted that the monocyte cytotoxin acts synergistically with actinomycin D in killing susceptible tumour cell lines (Matthews, 1981*b*). Cytotoxicity is enhanced and the cells are killed much earlier, cell death first being apparent about 8 h after exposure. In contrast to untreated cells, actinomycin D-treated L929 cells need be exposed to cytotoxin for as little as 1 h for near maximal cytotoxicity (Figure 3).

**Figure 3** Cytotoxicity to actinomycin D-treated L929 cells as a function of exposure time to monocyte cytotoxin. Cytotoxin was added to the cells at 0 h and removed at various times thereafter either by washing ×3 (▲) or by the addition of a neutralizing amount of cytotoxin antibody (▼). The assay was terminated at 20 h and actinomycin D at 1 $\mu$g ml$^{-1}$ was present throughout.

The major effect of actinomycin D is to inhibit transcription and ultimately protein synthesis. Other agents which inhibit protein synthesis were also tested for synergy with the monocyte cytotoxin. These agents were tested at concentrations spanning their minimal growth inhibitory concentration. No reproducible synergistic effect was found with puromycin or cycloheximide or with the antibiotics chloramphenicol or tetracycline which selectively inhibit mitochondrial protein synthesis (Figure 4).

*Effect on cell respiration*

Granger *et al.* (1980) reported that the cytostatic effect of mouse macrophages on tumour cells was mediated by inhibition of tumour cell respiration. The tumour cells attempted to compensate by increasing glycolysis. Does the monocyte cytotoxin induce similar changes in L929 cells?

L929 cells were exposed to cytotoxin for 24 h at 37°C before measurement of their respiration rate by an oxygen electrode. The respiration rate of cytotoxin-treated cells was 1.44 $\mu$l h$^{-1}$ per $10^6$ cells compared with 2.28 $\mu$l h$^{-1}$ per $10^6$ cells for untreated cells. Further, in cytotoxin-treated cells there was increased lactic acid production in 4/5 experiments (Table IV) as well as enhanced glucose uptake (Table IV). One possible interpretation of these data is that the cytotoxin causes mitochondrial dysfunction and the cells attempt to compensate with an increased rate of glycolysis.

**Figure 4** Synergy between monocyte cytotoxin and inhibitors of protein synthesis. L929 cells at $3 \times 10^5$ ml$^{-1}$ were incubated for 24 h with the inhibitor in the presence (▲) or absence (▼) of a subtoxic amount of cytotoxin.

**Table IV** Lactic acid production and glucose uptake by untreated and cytotoxin-treated L929 cells

| *Experiment No.* | *Cytotoxin present* | *Lactic acid* production* ($nmol\,l^{-1}$) | *Glucose uptake†* ($cpm\,mg^{-1}$ *protein*) |
|---|---|---|---|
| 1 | No | 0.991 ± 0.007 | 31,747 ± 6778 |
| | Yes | 2.046 ± 0.043 | 56,398 ± 4505 |
| 2 | No | 0.983 ± 0.116 | 7,210 ± 530 |
| | Yes | 0.911 ± 0.130 | 30,540 ± 2490 |
| 3 | No | 0.515 ± 0.018 | 8,187 ± 435 |
| | Yes | 1.200 ± 0.094 | 15,238 ± 2347 |
| 4 | No | 0.723 ± 0.140 | N.D.‡ |
| | Yes | 0.947 ± 0.021 | |
| 5 | No | 0.181 ± 0.130 | N.D. |
| | Yes | 0.506 ± 0.072 | |

*Lactic acid produced in the first 24 h after exposure to 1/20 dilution of cytotoxin.

†Measured after 24 h exposure to cytotoxin.

‡N.D. = not done.

### Discussion

The monocyte cytotoxin acts directly on the tumour target cells and needs to be in contact with them for at least 16–24 h. After 24 h exposure to the cytotoxin, the cells have a decreased respiration rate suggesting mitochondrial dysfunction but exhibit enhanced lactic acid production and glucose uptake indicating increased glycolysis. With small amounts of cytotoxin the cell may be able to compensate by generating sufficient energy by glycolysis and cytostasis ensues; larger amounts of cytotoxin may cause a more profound depression in respiration and failure of the cell to compensate results in cytotoxicity.

Addition of anti-cytotoxin antibody to cells exposed to the cytotoxin for upwards of 24 h can inhibit the effects of the cytotoxin. The accessibility to cytotoxin suggests that the cytotoxin must have remained on the cell surface. The failure of susceptible cells to adsorb cytotoxin from solution is not too surprising and indeed similar experiments with interferon were unsuccessful until highly purified, radioactively-labelled interferon became available (Aguet, 1980).

One possible mechanism for a cell surface action is that the cytotoxin prevents uptake by the cell of a nutrient, essential perhaps for mitochondrial function. The cytotoxin may act by competitively inhibiting uptake of the nutrient by the cell surface receptor; alternatively the cytotoxin may be an enzyme which attacks the cell surface receptor. The observation that the efficacy of the cytotoxin is inversely proportional to the cell concentration is consistent with both models. However, the trace concentration of the cytotoxin makes competitive inhibition the least likely alternative. There are some data suggestive of an enzymic action for the cytotoxin. Firstly the slope of the dose response curve is consistent with an enzyme model. Secondly, the cytotoxin is inhibited by phenanthroline, a metal chelator which inhibits many metallo-enzymes. It can be argued that the synergy between the cytotoxin and actinomycin D is due to the inhibition of protein synthesis by actinomycin D, thus preventing resynthesis of damaged cell surface receptors. However, there is probably an alternative explanation because puromycin and cycloheximide, which have a more direct action on protein synthesis than actinomycin D, do not act synergistically with the cytotoxin.

Previously we have noted the similarities between the human monocyte and rabbit TNF in terms of specificity, molecular weight on gel-filtration and mode of production and suggested that the monocyte cytotoxin may be the human analogue of rabbit TNF (Matthews, 1981*b*). In the work described here further similarities are apparent. Like the human cytotoxin, the interaction of rabbit TNF with L929 cells exhibits a lag period before cellular effects are obvious, a requirement for prolonged factor-cell contact, temperature dependenct (Matthews & Watkins, 1978; Ruff & Gifford, 1981*a*), enhancement by actinomycin D and depression by lowered serum concentration in the culture medium (Ruff & Gifford, 1981*a*). In addition, rabbit TNF is inhibited by phenanthroline (Ruff & Gifford, 1981*b*). The longer lag period for the human monocyte cytotoxin may be explained by its lower potency compared with rabbit TNF; with the latter, longer lag periods are noted with higher dilutions.

Because of the increased potency of rabbit TNF a more pronounced effect on cellular respiration might be expected. We have found that this is indeed the case and furthermore, electron microscopy of L929 cells treated with rabbit TNF has revealed that the first sign of cellular abnormality is manifest in the mitochondria, which become enlarged, translucent and exhibit fewer cristae.

Although the mode of action of the cytotoxin is far from proven, the following may serve as a working hypothesis for further studies. The monocyte cytotoxin is a metalloenzyme which attacks a cell surface receptor for a nutrient, essential perhaps for mitochondrial function.

I thank Mrs, M.L. Neale for capable technical assistance and Mr. D. Grimshaw for help with the oxygen electrode. The work was supported by a grant from the Cancer Research Campaign.

### References

ADAMS, D.O., JOHNSON, W.J., FIORITO, E. & NATHAN, C.F. (1981). Hydrogen peroxide and cytolytic factor can act synergistically in effecting cytolysis of neoplastic targets. *J. Immunol.*, **127**, 1973.

ADAMS, D.O., KAO, K.J., FARB, R. & PIZZO, S.V. (1980). Effector mechanisms of cytolytically activated macrophages. II. Secretion of a cytolytic factor by activated macrophages and its relationship to secreted neutral proteases. *J. Immunol.*, **124**, 293.

AGUET, M. (1980). High affinity binding of $^{125}$I-labelled mouse interferon to a specific cell surface receptor. *Nature*, **284**, 459.

CURRIE, G.A. (1978). Activated macrophages kill tumour cells by releasing arginase. *Nature*, **272**, 758.

MÄNNEL, D.N., MOORE, R.N. & MERGENHAGEN, S.E. (1980). Macrophages as a source of tumoricidal activity (Tumor necrotizing factor). *Infect. Immunol.*, **30**, 523.

MATTHEWS, N. (1978). Tumour necrosis factor from the rabbit. II. Production by monocytes. *Br. J. Cancer*, **38**, 310.

MATTHEWS, N. (1981*a*). Tumour necrosis factor from the rabbit. V. Synthesis *in vitro* by mononuclear phagocytes from various tissues of normal and BCG-injected rabbits. *Br. J. Cancer*, **44**, 418.

MATTHEWS, N. (1981*b*). Production of an anti-tumour cytotoxin by human monocytes. *Immunology*, **44**, 135.

MATTHEWS, N. (1982*a*). Production of an anti-tumour cytotoxin by human monocytes: comparison of endotoxin, interferons and other agents as inducers. *Br. J. Cancer*, **45**, 615.

MATTHEWS, N. (1982*b*). Human monocyte killing of tumour cells. Contribution of an extracellular cytotoxin. In Macrophages and natural killer cells, *Adv. Exp. Med. Biol.*, **155**, 721.

MATTHEWS, N. (1983). Effect on human monocyte killing of tumour cells of antibody raised against an extracellular monocyte cytotoxin. *Immunology*, **48** (in press).

MATTHEWS, N. & WATKINS, J.F. (1978). Tumour necrosis factor from the rabbit. I. Mode of action, specificity and physicochemical properties. *Br. J. Cancer*, **38**, 302.

NISSEN-MEYER, J. & HAMMERSTRØM, J. (1982). Physicochemical characterization of cytostatic factors released from human monocytes. *Infect. Immunol.*, **38**, 67.

READ, S.M. & NORTHCOTE, D.H. (1981). Minimization of variation in response to different proteins of the Coomassie blue G dye-binding assay for protein. *Anal. Biochem.*, **116**, 53.

RUFF, M.R. & GIFFORD, G.E. (1981*a*). Rabbit tumor necrosis factor: mechanism of action. *Infect. Immun.*, **31**, 380.

RUFF, M.R. & GIFFORD, G.E. (1981*b*). Tumor necrosis factor. In *Lymphokine Reports*, Vol. 2. (Ed. Pick) New York: Academic Press, p. 235.

WHITE, M.K., BRAMWELL, M.E. & HARRIS, H. (1981). Hexose transport in hybrids between malignant and normal cells. *Nature*, **294**, 232.